# Is perioperative antibiotic prophylaxis in the case of routine surgical removal of the third molar still justified? A randomized, double-blind, placebo-controlled clinical trial with a split-mouth design

**DOI:** 10.1007/s00784-022-04597-5

**Published:** 2022-07-06

**Authors:** Barbara Kirnbauer, Norbert Jakse, Astrid Truschnegg, Ivana Dzidic, Khaled Mukaddam, Michael Payer

**Affiliations:** 1 Department of Dental Medicine and Oral Health, Division of Oral Surgery and Orthodontics, Medical University of Graz, Billrothgasse 4, 8010 Graz, Austria; 2 Department of Oral Surgery, University Center for Dental Medicine Basel (UZB), University of Basel, Mattenstrasse 40, 4058 Basel, Switzerland

**Keywords:** Third molar surgery, Perioperative antibiotic prophylaxis, Oral surgery, Antibiotics

## Abstract

**Introduction:**

Since antimicrobial resistance, caused by various factors including antibiotic overuse and abuse, is a severe challenge, the necessity of perioperative antibiotic prophylactic for surgical third molar removal remains a contentious topic. This study determined whether perioperative antibiotic prophylaxis can reduce surgical site infections (SSIs), swelling, and pain in the case of surgical removal of wisdom teeth.

**Material and methods:**

A randomized, double-blind, placebo-controlled clinical trial with a split-mouth design. A study medication of 2 g amoxicillin, administered 1 h before the third molar removal, followed by 1.5 g each for the first 3 postoperative days, was compared with placebo medication. The primary outcome variable (SSI), secondary clinical parameters (swelling and trismus), and patient-centered outcome measures (bleeding, swelling, pain, and pain medication intake) were documented until postoperative day 7. Statistical analyses were done with a paired *t* test, *t* test for independent samples, Chi-square test, and McNemar test, including effect sizes.

**Results:**

Primary outcome SSI, in total 11%, and clinical parameters swelling and trismus were not significantly different between the two groups. The patient-centered outcome measures (bleeding, swelling, and pain) did not significantly differ, except for postoperative bleeding in the EG on day 0. No significant result was found with pain medication intake postoperative on days 0–7.

**Conclusions:**

Perioperative administration of oral antibiotics neither revealed additional benefits in patient-related outcome measures nor reduced postoperative complications compared with the placebo group indicated at routine surgical removal of noninflamed wisdom teeth.

**Clinical relevance:**

Taking antimicrobial resistance into account, clear recommendations for administering drugs, particularly antibiotics, are critical in oral surgery.

## Introduction 

Surgical removal of the third molar is one of the most common interventions in oral surgery [1]. Most young and healthy persons undergo this procedure for prophylactic reasons. With an incidence of up to 30%, postoperative complaints occur quite frequently [2–4]. At least for the first 5–7 days after surgery, edema, trismus, pain, and inflammation harm the well-being and quality of life of the otherwise healthy persons [5]. Especially, surgical site infections (SSIs) can be uncomfortable for a patient and cause long sick leaves and additional costs for the health care system [5, 6]. Several preventive measures have been described in the literature. Besides cryotherapy, antibacterial mouthwashes, topical gels, steroids, and anti-inflammatory drugs, routine administration of antibiotics is widespread [7]. However, as concluded in a recent review [8], there is no agreement on whether perioperative antibiotic prophylaxis should be administered for regular surgical removal of the third molar.

Worldwide drug misuse and overuse are some of the reasons we are currently facing AMR (antimicrobial resistance) [9, 10]. The declining efficacy of antimicrobial medication has become a reality in the form of superbugs, such as methicillin-resistant *staphylococcus aureus* or extremely drug-resistant tuberculosis [9, 11]. Every year, 700,000 people die due to a resistant infection, with 214,000 of them being newborns [9]. Concerning resistances, gram-positive and gram-negative organisms pose an exceptionally high risk because of their growing insensibility against ß-lactam antibiotics [9] and carbapenems [12]. As a consequence, the World Health Organization (WHO) defined a “Global action plan on antimicrobial resistance” with a focus on five strategic objectives: the improvement of awareness and understanding of AMR, increase in surveillance and research, decrease in the incidence of infections, pursuit of sustainable financing, and optimization of the application of antimicrobial drugs [13]. Referring to the latter WHO target, this study focuses on the necessity of antibiotics in the field of oral surgery.

With regard to supporting the responsible handling of antibiotic administration, the present study determined the noninferiority of a placebo medication in conventional surgical removal of the noninflamed wisdom teeth, while focusing on SSIs, swelling, trismus, and the patient’s subjective well-being compared with perioperative antibiotic prophylaxis with amoxicillin.

## Materials and methods

### Study design

**Fig. 1:**
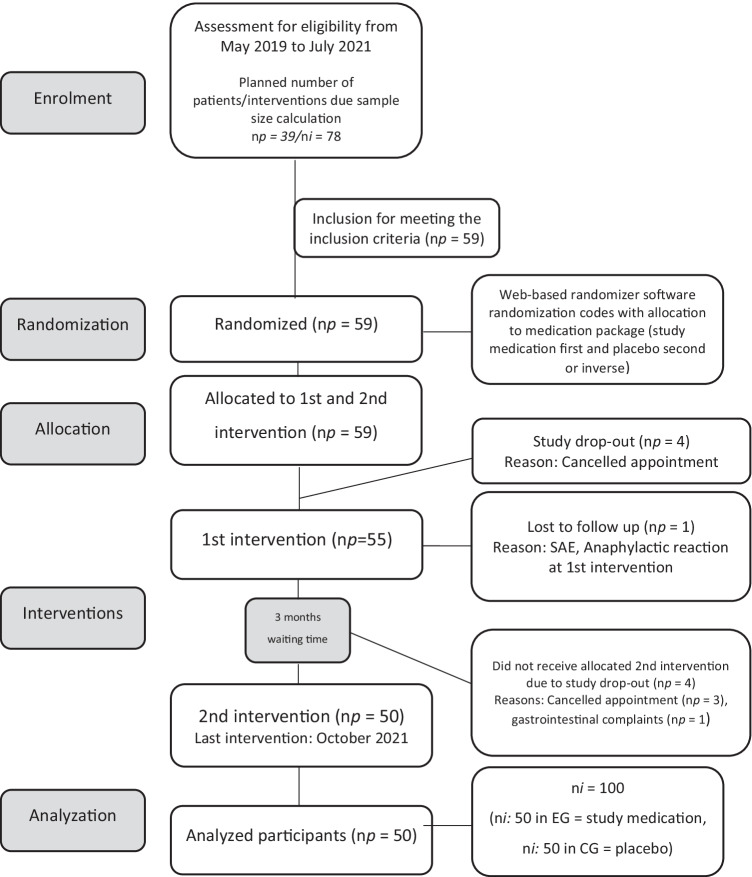
CONSORT flow diagram showing the participant enrolment, with the number of participants randomized and the randomization process allocated to both interventions, dropouts, reasons for dropouts, and the number analyzed for outcome measures (SAE, serious adverse event; np, number of patients; ni, number of interventions; EG, event group; CG, control group)

This study was conducted as a randomized, double-blinded, placebo-controlled single-center trial in a split-mouth design (Fig. 1). It was performed following the principles of the Declaration of Helsinki. Ethical approval was obtained from the ethical review committee (review board number 30–204 ex 17/18) and registered in the EudraCT database (number: 2017–004,986-8) at a university hospital. The study was accompanied and monitored by a local coordination center of clinical trials.

### Sample size calculation

A priori sample size calculation was performed for the target SSI (local wound infection or abscess with purulent secretion) with an online tool (Select Statistical Services Ltd, Exeter, UK) based on the experience in the literature. In the case of antibiotic administration, 0–5% inflammations were formerly detected (0% [14], 1% [15], 2.7%, and 5.5% [16]), and without an antibiotic medication, 5–15% (12.5% [14], 12.9% [17], 14.8% [4], 16% [16]) were reported. The mean difference in the prevalence was 14.1% [14, 17]. With a confidence interval of 95%, statistical power of 80%, and the assumption that the amount of infections is 2.5% in the antibiotic group and 15% in the placebo group, the necessity of recruiting 39 patients for 78 interventions was given. Therefore, 110 observations were planned. Unfortunately, there were 9% dropouts; thus, a final number of 50 participants with 100 surgeries was reached.

### Participant recruitment and eligibility criteria

Patients aged 16 years or older who were referred for surgical removal of four impacted or slightly impacted wisdom teeth between May 2019 and July 2021 were considered participants in this study. Inclusion criteria were as follows: four impacted or partially impacted third molars (18, 28, 38, 48) of average degree of difficulty; absence of actual local infection; normal state of health (American Society of Anesthesiologists classification, ASA 1); non or light smoker (< 10 cigarettes/day); absence of allergies or intolerances to local anesthetics, amoxicillin, or penicillin; no use of antibiotics within the previous 3 months; a lack of factors negatively influencing soft tissue healing and bone metabolism (e.g., antiresorptive medication, head, and neck radiotherapy); and no pregnancy and breastfeeding in female participants. Patients with general contraindications to wisdom tooth extraction surgery and those who did not meet the above criteria were excluded.

### Randomization, allocation, and blinding

Participants received written and verbal information about the surgical intervention and the clinical trial procedure. Participants who had signed the informed consent form were allocated to a sequential subject identification (ID) by the principal investigator. The participants were randomly assigned to their treatment ID and subsequent blinded medication package (two containers each with first event group, EG; second control group, CG; or inverse) using randomization codes generated by the web-based randomizer software [18]. A person not involved in the clinical procedure was responsible for strictly keeping the blinded medication packages and the allocation list locked. As a result, the surgeons, patients, and postoperative assessors were blinded. To unblind the pharmacy’s packaging list, the treatment ID list and patient ID list had to be combined.

### Clinical procedures

At the first study visit, general clinical parameters were assessed with a standardized health questionnaire. Patients were checked using panoramic radiography to radiological inclusion criteria of four (partially) impacted third molars of medium degree of difficulty according to the classification of Pell and Gregory [19] and Winter [20] for lower wisdom teeth. A cone-beam computed tomography scan was performed with a close association between the roots and the inferior alveolar nerve canal. Additionally, all patients underwent a radiation-free face scan for digital surface imaging with the same device (Planmeca ProMax 3D Max; Planmeca Oy, Helsinki, Finland). Furthermore, assessment of the maximum interincisal distance, as well as an analog face measurement with a tape measure (lateral corner of the eye–jaw angle; tragus–lateral corner of the mouth, tragus–pogonion, summarized in millimeter, mm), was performed as preoperative baseline values.

An hour before the start of wisdom tooth removal on the day of the first and second surgery, all patients received 40 mg of methylprednisolone (Urbason 40 mg, Sanofi-Aventis, Frankfurt am Main, Germany) orally and their study medication (26 hard gelatin capsules in a resealable container). The study medication was prepared at the local hospital pharmacy using Amoxilan 1000-mg tablets (G.L. Pharma GmbH, Lannach, Austria), newly packaged into hard gelatin capsules containing 250 mg amoxicillin each. Eight capsules were taken immediately, and on the following 3 days, six capsules (3 × 2 every 8 h). The EG received 250 mg amoxicillin per capsule (2 g amoxicillin on the day of surgery, 1.5 g amoxicillin on each on the following 3 days), while the CG received capsules filled with pharmacological inactive lactose monohydrate as placebo medication. The hospital pharmacy delivered two containers for each treatment ID with allocation to the first and second intervention that each patient randomly received both the amoxicillin and placebo once in varying order. A regimen (amoxicillin vs. placebo) was already applied in a recently published study at the study center [21]. Dexibuprofen (400-mg Seractil forte film-coated tablets, Gebro Pharma, Fieberbrunn, Austria) was prescribed, three times daily.

Three well-experienced oral surgeons performed the third molar surgery under strict hygiene guidelines in a surgical room, including sterile surgical laundry, sterile gloves, and preoperative facial wash of the patient. Each procedure followed a standardized protocol. This includes preoperative mouth rinse (Listerine cool mint, Johnson and Johnson, New Brunswick, New Jersey) for 1 min, followed by local infiltration and block anesthesia of the inferior alveolar, buccal, and palatal nerve with articaine and epinephrine 1:100,000 (Ultracain dental forte; Normon S.A. Tres Cantos, Madrid, Spain). First, the upper third molar was removed with elevators after full-thickness mucoperiosteal flap elevation, reflection, and osteotomy. Second, a full-thickness mucoperiosteal envelope flap was built at the lower jaw after incision (blade no. 15) along the ramus with lateral extension from the second molar. Osteotomy and, if necessary, tooth section were performed using a surgical handpiece with descending round burs and a conical mill under continuous sterile cooling liquid.

Tooth removal was done with elevators, followed by rinsing with sterile physiological saline solution and applying two gelatin sponges (Spongostan Dental; Johnson and Johnson, New Brunswick, New Jersey). Soft tissue wound closure was performed in both jaws with non-resorbable atraumatic sutures 5–0 (Dafilon, B. Braun, Tuttlingen, Germany) without using any temporary drains. After surgery, the patients got detailed instructions concerning postoperative behavior, study medication intake, and daily self-assessment. In the event of an emergency, all patients were given contact information to call for advice 24 h a day, 7 days a week. A minimum interval of 3 months between the first and second surgery was observed to prevent influence by the active ingredient.

### Clinical evaluation and data collection

Patients were reordered on postoperative day 1 (d 1) and day 7 (d 7), in concordance with Jakse et al. [22] for follow-up, including medication compliance, digital face scan, analog face measurements (swelling: lateral corner of the eye–jaw angle; tragus–lateral corner of the mouth, tragus–pogonion, summarized in millimeter; trismus: maximum interincisal distance, recorded in millimeter), and intraoral clinical investigation concerning potential SSIs. Regarding previously published data [23], SSI was defined as local inflammation, indicating solely wound irrigation, or the presence of an abscess, which required antibiotic treatment and incision and drainage with gauze. Alveolar osteitis was not recorded. Postoperative investigations were double-blinded by an experienced and trained staff different from the blinded surgeon.

For the digital analyses of the swelling, face scan datasets were exported in STL format (standard tessellation language) and imported into the coDiagnostiX software (Dental Wings GmbH, Chemnitz, Germany). To measure the volume of swelling, measurements were superimposed using the preoperative and both postoperative (d 1; d 7) scans using stable anatomic landmarks, such as the forehead, bridge, tip of the nose, and both eye sockets. Afterwards, the volume between preoperative and first postoperative (d 1) as well as preoperative and second postoperative (d 7) scans at both sides was segmented manually within the coDiagnostiX software slice by slice at a voxel size of 400 µm and resembled as the volume in milliliter (ml).

For the analysis of patient-centered outcomes, the bleeding, swelling, and pain parameters were postoperatively self-assessed from days 0–7 and documented on a 10-cm visual analog scale (VAS) extending from 0 (no pain) to 10 (very severe pain). Furthermore, the need for additional pain medication was self-documented dichotomously (yes = Y/no = N) until postoperative day 7.

### Statistical analysis

The primary outcome variable was the occurrence of SSIs, defined as the occurrence of local inflammation with edema and wound secretion or an abscess with purulent secretion as dichotomous measurement (Y/N) at postoperative day 7. Other secondary outcome variables were the clinical parameters of swelling (analog and digital), trismus (interincisal distance), and the patient-centered outcomes (bleeding, swelling, pain, and pain medication intake).

Descriptive and explorative analyses followed. For the primary outcome variable, Fisher’s exact test was performed. For the analog swelling analyses, tape measurements from the three time points (preoperative, day 1, and day 7) were summarized. Swelling volumes on days 1 and 7 were calculated for the digital swelling analyses. For the analyses of trismus, the decrease and increase of mouth opening as the difference between the interincisal distances of first follow-up (d 1) and preoperative time point and the second (d 7) and first follow-up were calculated. Paired *t* test was performed on the swelling and trismus variables. A *t* test for independent samples was applied for the patient-centered outcome variables (bleeding, swelling, and pain). The McNemar test was applied for the patient-centered outcome variable (pain medication intake). The Chi-square test was performed for the third molar classification (Table 1). Significance was set at alpha = 0.05. A *p* value < 0.05 was considered significant. Confidence interval of 95% (CI 95%) was calculated for clinical parameters swelling and trismus. Effect sizes for the outcome variables resembled Cohen’s d and phi coefficient. All analyses were performed with the SPSS software (IBM SPSS statistics 27.0, IBM Corporation, New York, NY).

**Table 1:** Patients’ baseline characteristics, third molar classification, and allocation of surgeons

		*n*	%	Min	Max	Mean	SD
Age	Female	29	58	16	26	21.2	3.3
	Male	21	42	16	27	21.3	3.1
	Total	50	100	16	27	21.2	3.2
		*n*	%				
Smoking	Non-smoker	41	82				
	Light smoker	9	18				
			EG			CG	
			*n*	%		*n*	%
Classification Pell and Gregory [19]	Depth	A	6	12		6	12
		B	36	72		36	72
		C	8	16		8	16
	Ramus	1	8	16	*p* = 0.846	6	12
		2	41	82		43	86
		3	1	2		1	2
Classification Winter [20]	Angulation	Distal	1	2		1	2
		Horizontal	1	2		1	2
		Mesial	38	76	*p* = 0.176	33	66
		Vertical	10	20		15	30
Surgeon		1	40	80		38	76
		2	9	18		11	22
		3	1	2		1	2

*n*, number of cases; *EG*, event group; *CG*, control group. Age (minimum = min, maximum = max, mean, standard deviation = SD), sex, smoking habits, third molar classification (Chi-square test), and surgeons (1, 2, 3)

## Results

The experimental protocols were implemented as planned, with no modifications. The participant flow diagram at the different phases of the study design is observed in Fig. 1. Fifty patients with 100 interventions (split-mouth design: 50 interventions in EG, 50 interventions in CG) were included in the final analyses. The patients’ baseline characteristics are summarized in Table 1.

### Primary outcome variable

With regard to the primary outcome variable, an overall SSI rate (local inflammation or abscess) of 11% (*n* = 11/100 cases) occurred, which means that an inflammatory rate of 6% (*n* = 3) in the EG and 16% (*n* = 8) in the CG, with no significant difference occurred between the groups (*p* = 0.200; phi = 0.160). Abscesses with purulent secretion developed in two cases out of 100 observations, one in the EG and one in the CG (Table 2).

**Table 2:** Primary outcome variable SSI

	EG		CG			
	***N***	%	***n***	%	***p*** value*	Effect size**
Yes	3	6	8	16	*p* = 0.200	phi = 0.160
No	47	94	42	84		

SSI (surgical site infection including local infection or abscess) as a dichotomous measure in the event group (EG) and the control group (CG) at day 7; number (*n*), percentage (%), significance levels (*Fisher’s exact test), and effect size (**phi coefficient)

### Secondary outcome variables

**Fig. 2:**
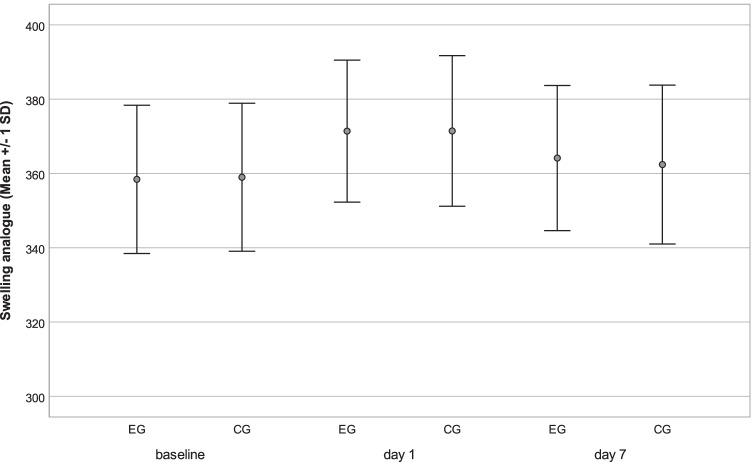
Secondary outcome variable swelling. Analog values by summarized tape measures in millimeters (lateral corner of the eye–jaw angle; tragus–lateral corner of the mouth, tragus–pogonion) were evaluated preoperatively (baseline) on a postoperative day 1 and day 7 in the event group (EG) and control group (CG). Resembled in mean with standard deviation (SD)

**Fig. 3:**
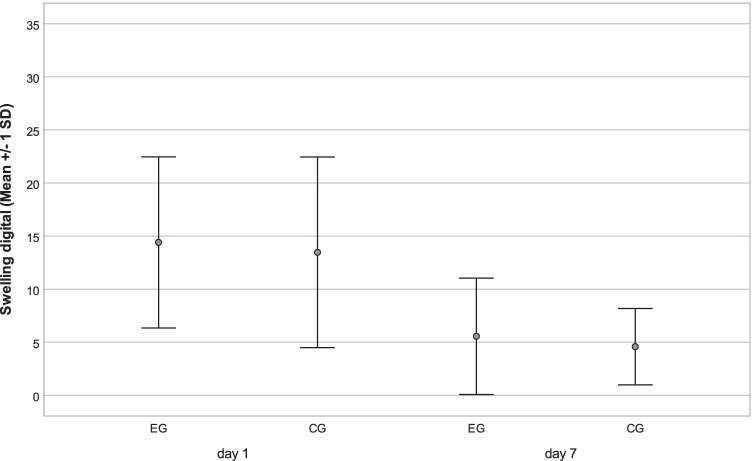
Secondary outcome variable swelling. Face scans were recorded preoperatively on postoperative day 1 and day 7. Digitally analyzed in milliliters via superimposition with preoperative scan on days 1 and 7 in the event group (EG) and control group (CG). Resembled in mean with standard deviation (SD)

**Fig. 4:**
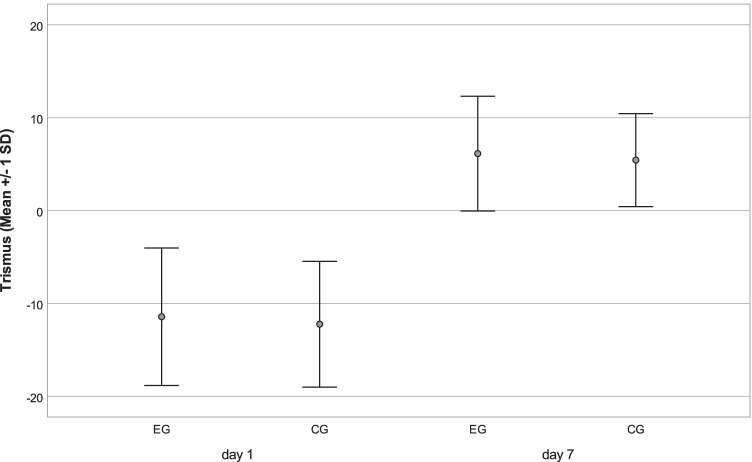
Secondary outcome variable trismus. The change of interincisal distance (in millimeter) between values measured preoperatively and on postoperative day 1 and between day 1 and day 7 in the event group (EG) and control group (CG). Resembled in mean with standard deviation (SD)

Concerning secondary outcome measures, first, the analog measurements of swelling reflected an increase on day 1 and a decrease until day 7 in both groups without a significant difference between them (*p* = 0.942; *p* = 0.574), whereas values on day 7 were slightly higher compared with the baseline measurements (Fig. 2; Table 3). Concerning the digital assessment of swelling, neither at the first nor the second postoperative appointment did the face scan evaluation show any significant difference between EG and CG (*p* = 0.727; *p* = 0.449), as shown in Fig. 3 and Table 3. The trismus parameter showed a similar trend with a decrease in the interincisal distance on day 1 and an increase until day 7 without a significant difference between EG and CG (*p* = 0.399; *p* = 0.570) (Fig. 4; Table 3).

**Table 3:** Secondary outcome variables

		EG		CG		
		mm (SD)	95%CI	mm (SD)	95%CI	Effect size**
		*p* = 0.782*				*d* = 0.05
Swelling analog	Baseline	358.46 (± 19.58)	352.6, 364.3	359.24 (± 19.57)	353.1, 364.8	
		*p* = 0.942*				*d* = 0.05
	d 1	371.9 (± 19.37)	365.8, 377.0	371.76 (± 19.79)	365.5, 377.4	
		*p* = 0.574*				*d* = 0.05
	d 7	363.47(± 19.74)	358.4–369.9	362.63(± 21.08)	356.1–368.7	
		ml (SD)	95%CI	ml (SD)	95%CI	
Swelling digital		*p* = 0.727*				*d* = 0.11
	d 1	14.79(± 8.09)	11.7, 17.1	13.87(± 9.07)	10.5, 16.5	
		*p* = 0.449*				*d* = 0.18
	d 7	5.47(± 5.08)	3.7, 7.4	4.59 (± 3.58)	3.4, 5.8	
		mm (SD)	95%CI	mm (SD)	95%CI	
		*p* = 0.399*				*d* = 0.13
Trismus	d 1 –baseline	− 11.29 (± 7.45)	− 13.6, -9.2	− 12.22 (± 6.90)	− 14.3, 10.2	
		*p* = 0.570*				*d* = 0.07
	d 7–d 1	5.94 (± 6.11)	4.3, 8.0	5.53 (± 4.91)	3.9, 6.9	

Swelling (analog: in millimeter = mm, digital: in milliliter = ml) and trismus (difference of maximum interincisal distance between time points in mm). Standard deviations (SD), 95%CI and significance level *p*, effect size (**d = Cohen’s d). Time points: preoperative (baseline), first follow-up (d 1), and second follow-up (d 7). *t test for paired samples

### Patient-centered outcome variables

**Fig. 5:**
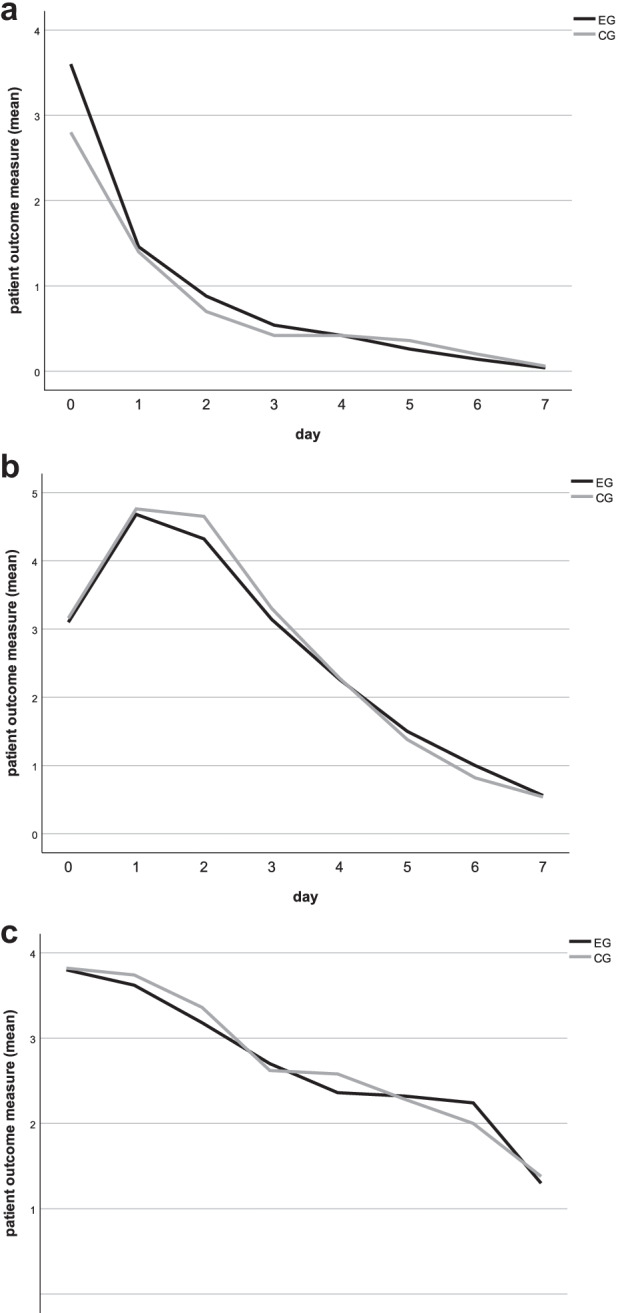
**a**–**c** Patient-centered outcomes bleeding, swelling, and pain. Mean values of postoperative bleeding, swelling, and pain on VAS from day 0 (day of surgery) until day 7. EG = dark, CG = light. **a** Day 0: significant difference between groups (*p* = 0.012). **b**–**c** No significant difference occurred by *t* test for independent samples

**Fig. 6:**
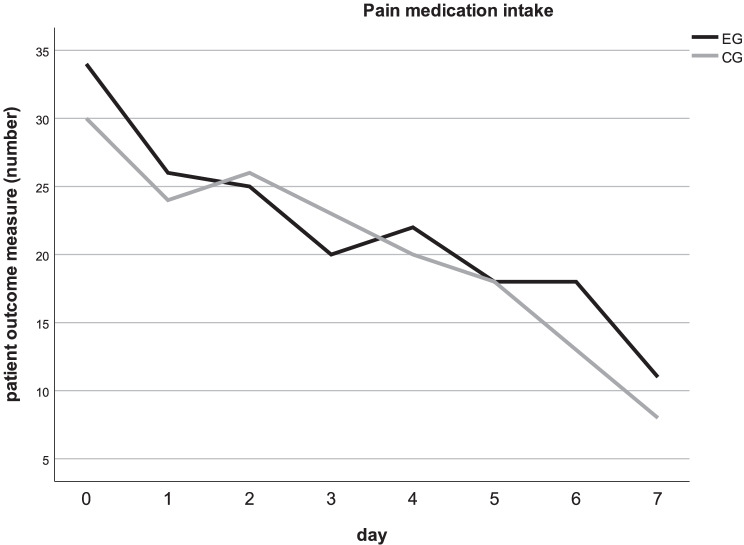
Patient-centered outcomes pain medication intake. The number of patients needing pain medication intake was evaluated postoperatively from day 0 (day of surgery) until day 7. EG = dark, CG = light. No significant difference occurred in the McNemar test

The three patient-centered outcome variables (bleeding, swelling, and pain) continuously decreased until postoperative day 7. However, a significant difference was observed with bleeding in the EG (day 0: *p* = 0.012) postoperatively (Fig. 5; Table 4). The self-assessment of pain medication intake resulted in no significant difference between the EG and CG at any postoperative time point (Fig. 6; Table 5).

**Table 4:** Patient-centered outcomes: bleeding, swelling, and pain

	Bleeding				Swelling				Pain			
	EG	CG			EG	CG			EG	CG		
	Mean (SD)	Mean (SD)	***p*** value*	Effect size**	Mean (SD)	Mean (SD)	***p*** value*	Effect size**	Mean (SD)	Mean (SD)	*p* value*	Effect size**
Day 0	3.60 (2.19)	2.80 (2.22)	*p* = 0.012	*d* = 0.18	3.10 (2.03)	3.16 (2.16)	*p* = 0.838	*d* = 0.01	*3.80 *(2.18)	*3.82 *(2.43)	*p =* 0.951	*d = *0.00
Day 1	1.46 (1.74)	1.40 (1.47)	*p* = 0.829	*d* = 0.02	4.68 (2.24)	4.76 (2.12)	*p* = 0.811	*d* = 0.02	*3.62 *(2.17)	*3.74 *(2.41)	*p = *0.723	*d = *0.03
Day 2	0.88 (1.21)	0.70 (1.05)	*p* = 0.345	*d* = 0.08	4.32 (2.19)	4.65 (2.08)	*p* = 0.295	*d* = 0.08	*3.18 *(2.24)	*3.36 *(2.38)	*p = *0.624	*d = *0.04﻿
Day 3	0.54 (1.05)	0.42 (0.88)	*p* = 0.479	*d* = 0.06	3.14 (2.01)	3.30 (1.75)	*p* = 0.577	*d* = 0.04	*2.70 *(2.00)	*2.62 (2.15)*	*p = *0.814	*d = *0.02
Day 4	0.42 (0.86)	0.42 (0.86)	*p* = 1.000	*d* = 0.00	2.26 (1.87)	2.28 (1.46)	*p* = 0.938	*d* = 0.01	*2.36 *(2.03)	*2.58 *(2.20)	*p = *0.553	*d = *0.05
Day 5	0.26 (0.69)	0.36 (0.83)	*p* = 0.451	*d* = 0.07	1.50 (1.69)	1.38 (1.23)	*p* = 0.629	*d* = 0.04	*2.32 *(2.19)	*2.28 *(2.06)	*p = *0.917	*d =* 0.01
Day 6	0.14 (0.45)	0.20 (0.57)	*p* = 0.554	*d* = 0.07	1.00 (1.36)	0.82 (0.96)	*p* = 0.415	*d* = 0.08	*2.24 *(1.93)	*2.00 *(2.23)	*p = *0.480	*d = *0.06
Day 7	0.04 (0.20)	0.06 (0.31)	*p* = 0.709	*d* = 0.04	0.56 (0.95)	0.54 (0.71)	*p* = 0.890	*d* = 0.01	1.30 (1.40)	1.38 (1.93)	*p = *0.735	*d = *0.02

Mean values (mean), standard deviations (SD), significance levels *p*, and effect size (**Cohen’s *d*) in the event group (EG) and the control group (CG) from day 0 until day 7. *t test for independent samples. No significance except bleeding on day 0

**Table 5:** Patient-centered outcome: pain medication intake

	Pain medication intake			
	EG	CG		
	*n*	*n*	*p* value*	Effect size**
Day 0	34	30	*p* = 0.424	0.17
Day 1	26	24	*p* = 0.774	0.08
Day 2	25	26	*p* = 0.999	0.04
Day 3	20	23	*p* = 0.453	0.12
Day 4	22	20	*p* = 0.791	0.08
Day 5	18	18	*p* = 0.999	0.00
Day 6	18	13	*p* = 0.227	0.22
Day 7	11	8	*p* = 0.508	0.15

Number (*n*) of patients with the need for pain medication intake, significance levels *p*, and effect size (**phi coefficient) in the event group (EG) and the control group (CG), evaluated postoperatively from day 0 (day of surgery) until day 7. *McNemar test. No significant difference occurred

## Discussion

In this investigation, neither a statistically significant evidence nor a clinically relevant difference was found to underline the superiority of perioperative antibiotic prophylaxis for preventing postoperative infective events in the case of routine removal of impacted wisdom teeth without local inflammation.

The development of antimicrobials paved the way for the age of modern medicine. Their effectiveness has allowed the performance of life-saving medical key procedures during the last 8 decades, e.g., joint replacement, cesarean sections, gastrointestinal surgeries, or oncological and immune system suppressing interventions securely and successfully [24]. However, besides an increased number of medical procedures, an aging society, and poor sanitation and hygiene in low and middle-income countries, inadequate and frequent use of antimicrobials destabilize a supposedly safe system [9, 25]. Thus, this study was conducted in the sense of resistance during prophylaxis and to promote the waiver of antibiotics whenever possible. Due to ambiguity, it has to be mentioned that further clinical trials are reported to be necessary [8]. Several published studies identified effects which significantly speak for [4, 14, 23, 26] and against [15, 27–32] antibiotic prophylaxis. Indeed, the necessity is still given to perform prospective, randomized, placebo-controlled, and practical clinical trials [23] to find a clear consensus about prophylactic antibiotic administration for healthy patients [8]. It has to be highlighted that the number needed to treat to prevent one SSI is rather high and lies at 143, according to a cohort study by Lang et al. [23]. Alternatively, present guidelines still support a perioperative antibiosis [35, 44], whereas the decision suggests to stay with the surgeon and observe each patient’s risk [35]. Therefore, it should be mentioned when considering antibiotic administration that a differentiation between not inflamed, clean–contaminated, and inflamed or even dirty conditions, such as pericoronitis, fistulas, infected cysts, or purulent secretion, has to be performed not only for the prevention of systemic [45] but also for local wound healing complications [35, 44].

This present study attempted to decrease the bias as much as possible by using a randomized, double-blind, placebo-controlled study design in clean–contaminated conditions, as performed in other studies over the last decade [14, 15, 28, 30, 31]. With split-mouth design less frequently shown in the literature [15, 29], patients acted as their controls, resulting in a higher homogeneity within the study population. As mentioned previously [31], the risk of opposite medical interaction was minimized with the prohibition of antibiotic intake 3 months before the start and the same waiting time between the first and second interventions. Furthermore, the mean age of 21 years [29] resembles the featured phase of prophylactic third molar intervention [33]. Similar but slightly older age distribution can be found with 26.4, 23.0, and 28.5 years in other studies [23, 28, 31].

Further in this study, only upper and lower third molar surgery cases were included at two appointments (18, 48 vs 28, 38). Although this is in contrast to others [15, 28, 31], it resembles the routine protocol at the study center and as previously performed [29]. Impacted third molars had to be of medium difficulty, preferably class B and class 2, according to Pell and Gregory [19], to achieve a balanced degree of osteotomy and similar surgery duration among the study population. Regarding the knowledge that surgical trauma potentially leads to a higher degree of tissue injury followed by an increased “inflammatory response” [34], a limited selection of three experienced oral surgeons was allocated in this study.

Perioperative antibiotic prophylaxis is the administration of antibiotics before, during, or after a diagnostic, therapeutic, or surgical operation to prevent infectious adverse effects [35]. Therefore, antibiotics are not thought to replace good surgical techniques; rather, they should accompany interventional procedures [35]. Within the oral cavity, mainly mixed infections occur. The ideal antibiotic should be non-toxic, easy to apply, and broadly effective against gram-positive, gram-negative, and anaerobic bacteria, such as amoxicillin [15], which is the most widespread in Europe and still resembles the key antibiotic in medicine [10]. In case of incompatibility, cephalosporins and macrolides/lincosamides are frequently prescribed [36]. Thus, correlating with others [15, 28, 30, 31] and according to the common empirically based clinical approach [10], the choice in this study fell on preoperatively initiated amoxicillin, orally administered for 4 days, as described by Payer et al. [21]. Although concerning the active ingredient, Sayd et al. [37] did not find a significant difference between amoxicillin–clavulanic acid and azithromycin in 108 patients, similar to Adde et al. [27] at 71 participants in the comparison of amoxicillin with clindamycin. Equally, as Reiland et al. [38] did regarding the application in a retrospective cohort study of 1895 samples, analyzing the difference per oral and intravenous administration. Iglesias et al. [39] suggested the noninclusion of clavulanate due to a significantly higher rate of gastrointestinal complications (5.5%) (diarrhea from *Clostridium difficile* infection) and resistance promotion [10]. The pre- or postoperative start of the medication regimen is also discussed in the literature [41]. Although Lopez-Cedrun et al. [14] could not find a statistically relevant difference in a sample of 123 patients, including a parallel-group study, we relied on the sufficient plasma levels suggested by Allegranzi et al. due to a 60-min preoperative antibiotic administration [41]. Thus, former recommendations [40, 41] and the recently published studies in this field [15, 28, 29, 31] were followed. Generally, a maximum duration of 24 h in surgery and a single-shot antibiotic use with a possible additional intraoperative dose are recommended, which in particular, focused on the prevention of resistance development [8]. This contrasts with the prolonged protocol used in this study, which however resembles a general procedure in oral surgery [15, 28]. However, this issue should not be further contentious. This study provided another important jigsaw piece to prove the nonsuperiority of antibiotic prophylaxis, opposite to the placebo medication in clean–contaminated sites in routine wisdom tooth surgeries as previously noted [21].

In this study, the primary outcome variable was the presence of SSIs. From our perspective, SSI means the occurrence of local inflammation at the extraction site with cloudy secretion and a tendency of propagation or the presence of an abscess with purulent secretion. In both cases, a patient needs dental care. Local inflammations were treated with disinfecting local irrigation, while abscesses underwent an incision with gauze drainage and further antibiosis. Dry sockets were not included in our clinical investigation because this clinical picture shows neither the signs of extraoral swelling, fever, and trismus nor a purulent secretion and may not result in a life-threatening situation. Thus, they cannot be clinically investigated when flaps are closed completely. The definition of SSIs varies in different reports, including swelling, pain, increased body temperature, or c-reactive protein levels [15, 28, 42]. Therefore, besides different study designs, it is challenging to directly compare the results of this investigation with others. Overall, we identified an infection rate of 11% within our study population, which is higher than the reported 0.03% by Milani et al. [31], 1% by Xue et al. [15], 4% by Lopez-Celdrun et al. [14], and 5.7% by Lang et al. [23]. In this context, noting especially the studies concerned with the lowest values [15, 31], potential sources of bias should be discussed. On one hand, the sample size calculation was not described in detail and may be too low with 20–30 in each group, and on the other hand, postoperative assessment methods were not transparently described [15, 31]. Concerning the effect of antibiotics versus placebo in our study, no significant difference occurred between the two treatment groups. In detail, eight patients were recognized in the placebo group, which is in accordance with the works of Bezerra et al. (11.76%) [29], Lopez-Celdrun et al. (12.5%) [14], Artegoita et al. (12.9%) [17], Monaco et al. (14.82%) [4], and Lacasa et al. (16%) [16]. The three inflammations of our EG were also rather confirmed with the studies of Monaco et al. (3.12%) [4], Lang et al. (5%) [23], Lacasa et al. (5.3%) [16], and Milani et al. (6.2%) [31]. In the present trial, the infection rates seem to be multifactorial. First, only third molars without any sign of inflammation were removed to observe the specification of clean–contaminated situations on the day of surgery. Signs of inflammation change the conditions toward contaminated or even dirty conditions, which modifies the antibiotic regime [35, 44]. Second, the patients’ preparation with aseptic mouthwashes and measures against the progression of swelling, such as preoperatively administered corticosteroids and gentle intraoperative tissue management, may impact infection prophylaxis instead of perioperative antibiotic administration.

The nonsuperiority of the antibiotic regime also seems to be underlined by the presented results concerning trismus and swelling. Although in a study of 293 participants [30], perioperative antibiosis showed a statistically significantly lower amount of swelling compared to placebo, it was equally found that without antibiotic use, the swelling also significantly decreased until postoperative day 7 [42]. Furthermore, Xue et al. [15] reported no significant difference between amoxicillin and placebo administration 2 and 10 days (*p* = 0.110; *p* = 1.000) after the third molar surgery, as similarly done by Lacasa et al. [16] from the third postoperative day. Furthermore, there is no significant difference between EG and CG in analog face measurement and an additional digital comparison of face scans, with mean volumes of 14.79 ml versus 13.87 ml on postoperative day 1 and 5.47 ml and 4.59 ml on day 7. Regarding those parallel measurements, which to the best of our knowledge have never been performed in this form before, we believe we can provide an alternative digital method that is worth continuing in future investigations and sufficient evidence that speaks against the prophylactic use of antibiotics. The latter is the same underlined by trismus development, not reflecting any significant difference at any postoperative time point (d 1: *p* = 0.399; d 7: *p* = 0.570), as reported in an earlier RCT [31] at postoperative day 4 and 7.

Patient-centered outcomes are an essential source of information in medicine. In a recent review [5], pain after the third molar removal was highlighted as having the most influence on patients’ quality of life. Besides clinical parameters (pus secretion, swelling, and trismus), patients’ subjective impressions concerning bleeding, swelling, and pain were investigated. Neither swelling nor pain revealed any significant result between the EG and CG, which correlates with the course of the needed pain medication. Milani et al. [31] reported a similar finding with no significant difference in pain between the groups with antibiotics and placebo medication on postoperative days 4 and 7. An additional argument can be presented with this knowledge, and straightforward advice is possible when patients ask for antibiotic prophylaxis, believing in rapid recovery with less pain. From our point of view, these results also justify our opinion of antibiotic reluctance concerning alveolitis, as no difference occurred in the patient-related outcome measures, which were evaluated for postoperative pain. The only exception presented is the “bleeding” parameter on the day of surgery. There is evidence that antibiotics administered along with oral anticoagulants lead to an increased incidence of postoperative bleeding [43]. However, the effect in our study cannot be explained because only healthy subjects were included here. Thus, this parameter seems to need further investigation.

One minor limitation of this study is the overall dropout of 9 patients. Anaphylactic reactions and gastrointestinal complaints resembled the minority opposite, a lack of motivation to return. An 8.5% of patients were lost in the first study phase, whereas a further 6.8% missed the second intervention. Nevertheless, the calculated limit of necessary samples and surgeries was fulfilled, from which it is concluded to be able to present reliable results. Furthermore, compared with two other randomized clinical trials [28, 29], our case number (50 patients with 100 surgeries) exceeds that of Bezerra et al. [29]. They only included 34 patients in their split-mouth study, similar to Artegoita et al. [28]. At the expense of the total case number, but to the benefit of the unbiased split-mouth analysis, the decision was made to only include patients (number, *n* = 50) who had both interventions completed, resulting in 100 interventions (Fig. 1). Another limitation of this study might be the relatively short follow-up period of 7 postoperative days. The thought was to follow our routine clinical protocol [22] and to combine final check-ups and suture removal in one appointment to increase the patient’s compliance to return to follow-up. However, this comparatively short period might have influenced the low rate of SSIs. An infection may occur, in the case of delayed wound closure beyond the 7-day follow-up, which was not investigated in this study. Some studies described the usefulness of prolonged investigation periods of 10 days up to 8 weeks [14, 15, 28], whereas others have applied the same as in this study [30, 31, 37]. However, in our opinion, a delayed infection due to wound dehiscence may be induced by shifted food residues, which do not have a causal relationship to the effect of a perioperative antibiosis. For a third limitation, 26% of digital face scans were not usable. This happened due to technical problems (e.g., caused by motion artifacts or artifacts due to long beards and irritating hairstyles). As a result, cases where no superimposition was appropriate were ruled out. Nevertheless, the case number seems to be balanced with a lack of 13 in the EG and 13 in the CG. Although it could be shown that the application of this tool is more than equal to the analog measurement, the technical aspects, including an increased effort of time, equipment, and costs, should be considered.

## Conclusions

Regarding the parallel measurements of swelling, which to the best of our knowledge have never been performed in this form before, we believe that we can provide an alternative digital method that is worth continuing in future investigations and sufficient evidence that speaks against the prophylactic use of antibiotics. This study demonstrated that prophylactic perioperative antibiotic treatment is not preferable to a placebo medicine, based on objective clinical and subjective patient outcome data. This indicates that perioperative prophylactic antibiosis at routine surgical removal of third molars in clean–contaminated sites, where no sign of local inflammation is present, generally seems unnecessary, as far as advanced hygiene guidelines are observed and experienced surgeons guarantee gentle intraoperative tissue management within a short surgery time. To prevent overtreatment of patients and help reduce the worldwide consumption of antimicrobials, we suggest carefully weighing the individual risks and benefits in order to avoid antibiotics in such cases.
